# A spiking continuous attractor network for event-driven neuromorphic robot guidance

**DOI:** 10.3389/fnins.2026.1910808

**Published:** 2026-09-16

**Authors:** Muhammad Aitsam, Aung Htet, Syed Saad Hasan, Alejandro Jimenez Rodriguez, Alessandro Di Nuovo

**Affiliations:** 1 Smart Interactive Technologies Research Laboratory, School of Computing and Digital Technologies, Sheffield Hallam University, Sheffield, United Kingdom; 2 Department of Computer, Control, and Management Engineering (DIAG), Sapienza University of Rome, Rome, Italy; 3 Università degli Studi di Catania, Catania, Italy

**Keywords:** continuous attractor network, dynamic neural field, event-based vision, leaky integrate-and-fire, neuromorphic computing, real-time robot guidance, spiking neural network, SpiNNaker 2

## Abstract

Event cameras provide fast, sparse visual measurements, but their output alone does not supply the persistent state required for closed-loop tracking. This study presents a hybrid event-driven perception-to-action system in which a Prophesee GenX320 (Prophesee SA, Paris, France) event camera and a lightweight Raspberry Pi 5 (Raspberry Pi Ltd., Cambridge, UK) front end provide target evidence to a spiking continuous attractor network running on a single SpiNNaker 2 (SpiNNcloud Systems GmbH, Dresden, Germany) chip. Recurrent difference-of-Gaussians connectivity forms a localised activity bump whose decoded position guides a simulated robot in a hardware-in-the-loop task. The network design is grounded in Amari's neural-field framework, with local excitation and broader inhibition used to select a compact, self-sustaining operating regime. On hardware, the measured transition between collapsed, stable, and saturated states follows the predicted stability corridor across 96 parameter settings. At the deployed operating point, the bump remains active for at least 8 s after input removal and tracks moving event-camera input across three target speeds. Against a registered target trajectory, the recurrent estimate has a mean error of 5.65 lattice cells and is 15% less jittery than the host-smoothed estimate that drives it. The median end-to-end latency from the close of an event-accumulation window to the corresponding bump update is 8.4 ms. In closed-loop guidance with periodic sensor dropout, all 10 recurrent and all 10 matched non-recurrent trials reached the goal, whereas the recurrent condition reached it 1.50 s sooner on average. A complementary finite-grid convergence analysis shows that weak, deployed, and stronger inhibition conditions exhibit expansion, bounded compact dynamics, and contraction, respectively. These results demonstrate that a continuum-guided, finite-grid-validated spiking attractor can provide persistent on-chip state for event-driven robot guidance.

## 1 Introduction

Event cameras report asynchronous changes in log intensity with microsecond timing rather than producing synchronous image frames (Gallego et al., 2022). Their sparse output and immediate response to motion make them well-suited to low-latency robotic perception. Spiking neural networks on neuromorphic hardware are a natural processing substrate because both sensing and computation communicate through sparse events and can reduce energy use relative to frame-based, clock-driven pipelines (Davies et al., 2018; Mayr et al., 2019). This pairing, however, does not by itself solve the continuous state-estimation problem required for closed-loop tracking. A controller must maintain a stable estimate from noisy, intermittent evidence and update it coherently as the target moves.

Continuous attractor networks provide a recurrent mechanism for holding such an estimate (Khona and Fiete, 2022). In Amari's neural-field formulation, local excitation combined with broader inhibition supports a localised activity packet, or bump, on a topographic sheet (Amari, 1977). The bump location represents a continuous variable, persists when input is weak or temporarily absent, and shifts when the input moves. Although continuous attractors are commonly associated with internal variables such as head direction and spatial position (Khona and Fiete, 2022; Monaco et al., 2020), related bump dynamics also describe target selection in retinotopic structures such as the superior colliculus (Munoz and Istvan, 1998; Trappenberg et al., 2001; Marino et al., 2012). We adopt this computational principle rather than attempting to reproduce the full biological circuit: an event-camera front end supplies a target map to a recurrent spiking sheet, and the resulting bump provides the target estimate for movement control.

Existing event-driven tracking pipelines often compute a centroid or heat map from the sensor and retain state through host-side filtering. Such filtering can reduce noise, but it does not establish whether the neuromorphic device itself contains a recurrent state with a predictable operating regime. The distinction can be tested by removing the input and by measuring the boundary between collapse, stable localisation, and saturation. These tests identify the contribution of on-chip recurrence separately from the preprocessing that supplies its input.

Figure 1 shows the system developed here. A Prophesee GenX320 event camera and Raspberry Pi 5 front end drive a recurrent spiking sheet on one SpiNNaker 2 chip; only event preprocessing and the final motor mapping remain on the host. Previous event-driven systems on SpiNNaker have used earlier dynamic vision sensors with full-scale machines or dedicated interfaces (D'Angelo et al., 2022; Ghosh et al., 2022; Romero Bermudez et al., 2023), while reported closed-loop control on SpiNNaker 2 has used simulated sensory input (Arfa et al., 2025). The novelty of this study lies in the specific integration of a physical GenX320 sensor, a low-power edge front end, and a single SpiNNaker 2 chip running a continuum-guided recurrent attractor, together with finite-grid convergence analysis and measurements of persistence, operating-regime boundaries, tracking, and hardware-in-the-loop guidance.

**Figure 1:**
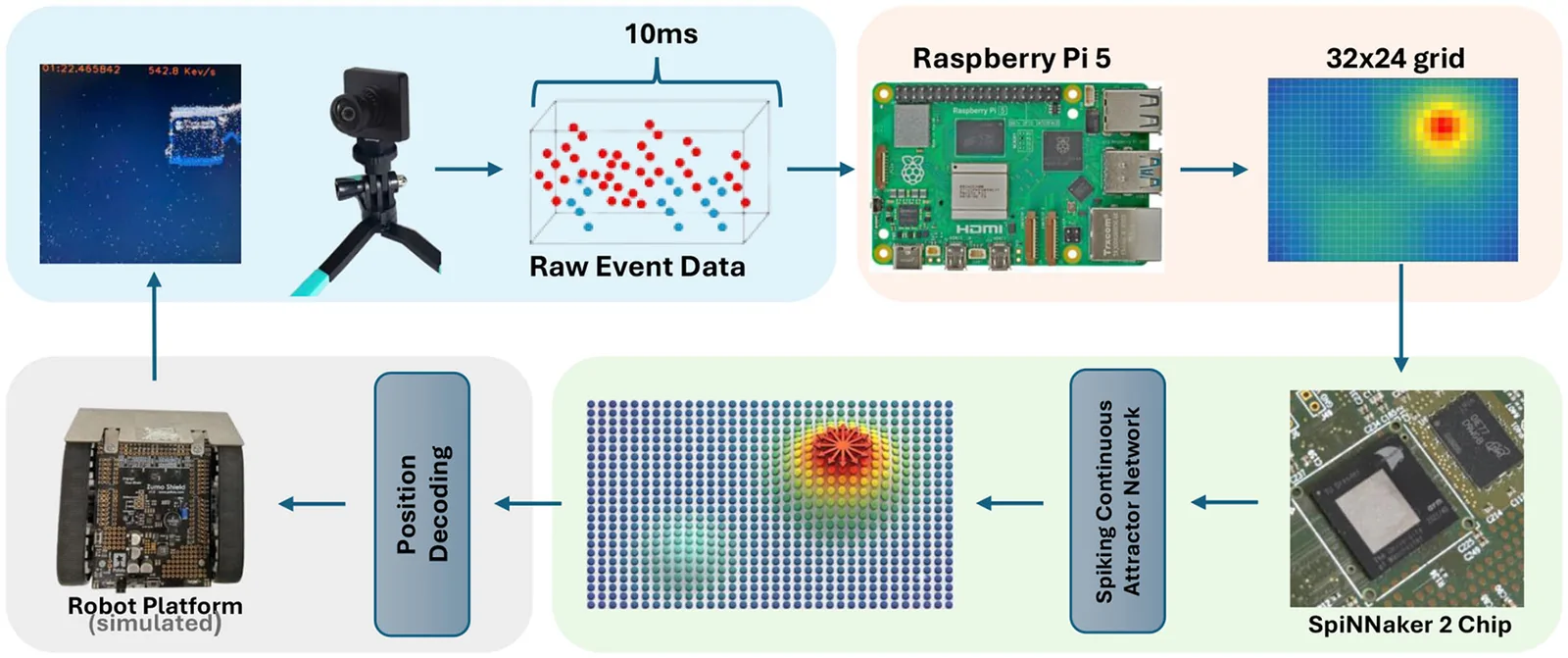
Complete closed-loop pipeline. The GenX320 event camera streams asynchronous events to the Raspberry Pi 5, which accumulates them over 10 ms windows, bins them onto a 32 × 24 grid, and transmits a target estimate to the SpiNNaker 2 chip. The on-chip spiking continuous attractor network forms a localised activity bump whose decoded position guides a simulated robot in a hardware-in-the-loop configuration.

The study makes three contributions:

• An end-to-end event-driven perception-to-action loop with the recurrent state resident on a single SpiNNaker 2 chip, sub-millisecond spike injection, and a median end-to-end latency of 8.44 ms;
• A neural-field design criterion for selecting the quantised recurrent kernel, complemented by direct finite-grid analysis of convergence, seed-size dependence, radial growth, and the sustained period-two state; and
• Hardware experiments that measure the stability corridor, demonstrate at least 8 s of persistence without input, separate the effect of recurrence from host preprocessing, and use the decoded bump for closed-loop robot guidance.

Section 2 develops the theoretical framework and maps it to the finite quantised network. Section 3 describes the physical system and experimental protocol. Section 4 reports the hardware results, and Sections 5 and 6 discuss their implications and conclude the study.

## 2 Theoretical framework

We formulate the tracking network as a discretised dynamic neural field and use Amari's framework (Amari, 1977) to identify a regime in which local excitation sustains a compact activity bump while broader inhibition contains it. The same formulation links the event-camera input, the recurrent state, and the decoded position used for guidance. We first define the spiking sheet, then derive continuum design criteria, examine the finite quantised network directly, and finally map the selected parameters to hardware.

### 2.1 Spiking network model

The recurrent sheet is implemented as a *W*×*H* lattice with *W* = 32 and *H* = 24, giving *N* = *WH* = 768 neurones. Each grid cell (*i, j*) maps to one neurone through the topographic index:


$$\begin{array}{l} n\left(i,j\right)=jW+i,\;i\in \left\{0,\ldots ,W-1\right\},\;j\in \left\{0,\ldots ,H-1\right\}. \end{array}$$ (1)

Equation 1 defines this one-to-one mapping keeps the event-camera representation, the spiking. sheet, and the tracking readout in the same spatial coordinate system.

Neurone *n* follows discrete-time leaky integrate-and-fire dynamics,


$$\begin{array}{l} V_{n}\left(t+\text{Δ}t\right)=\alpha V_{n}\left(t\right)+\overset{N}{\underset{m=1}{\sum }}W_{nm}s_{m}\left(t\right)+I_{n}\left(t\right), \end{array}$$ (2)

where *V*_*n*_ is the membrane potential, α is the leak factor, *s*_*m*_(*t*) ∈ {0, 1} indicates whether neurone *m* spiked at time *t*, *W*_*nm*_ is the recurrent weight from neurone *m* to neurone *n*, and *I*_*n*_(*t*) is the feedforward input from the event-processing front end. A spike is emitted when *V*_*n*_ crosses the threshold ϑ, after which the membrane potential is reset to *V*_reset_.

For interpretation, the leak can be written as the effective membrane time constant:


$$\begin{array}{l} \tau_{m}=\frac{\text{Δ}t}{1-\alpha }. \end{array}$$ (3)

With Δ*t* = 1ms and the deployed value α = 0.5, Equation 3 gives τ_*m*_ = 2ms. The short time constant is intentional: the sheet responds quickly to new events while still allowing recurrent activity to persist briefly when the input is sparse or intermittent.

The recurrent weights implement a spatially symmetric difference-of-Gaussians (DoG) interaction:


$$\begin{array}{l} W_{nm}=w\left(d_{nm}\right),\;w\left(r\right)=A_{e}e^{-r^{2}/2\sigma_{e}^{2}}-A_{i}e^{-r^{2}/2\sigma_{i}^{2}}, \end{array}$$ (4)

where *d*_*nm*_ = ||**r**_*n*_−**r**_*m*_|| is the lattice distance between neurones. The first term gives short-range excitation, and the second term gives a broader inhibitory surround, with σ_*e*_ < σ_*i*_. On hardware, this kernel is radially truncated and quantised to integer weights. Conceptually, the kernel makes nearby cells support one another while suppressing more distant activity, which is the mechanism that produces a compact activity bump rather than either isolated single-cell activity or uncontrolled spread.

### 2.2 Connection to Amari's neural field

Amari's neural field model describes how activity evolves in a continuous sheet of interacting neural units. In that framework, the state variable *u*(**x**, *t*) represents the activation at position **x**, the function *f*(*u*) converts activation into output activity, and the connection kernel *w*(||**x**−**x**′||) describes how activity at one location influences activity elsewhere (Amari, 1977). The corresponding continuum description of the implemented spiking sheet is


$$\begin{array}{l} \tau_{m}\frac{\partial u\left(\text{x},t\right)}{\partial t}=-u\left(\text{x},t\right)+\int_{\text{Ω}}w\left(||\text{x}-{\text{x}}^{\prime }||\right)H\left(u\left({\text{x}}^{\prime },t\right)-\vartheta \right)d{\text{x}}^{\prime }+I\left(\text{x},t\right). \end{array}$$ (5)

Here *H*(·) is a thresholded firing-rate approximation to the spiking output, *I*(**x**, *t*) is the event-camera input projected onto the sheet, and the kernel *w* has the same local-excitation, surround-inhibition form as the implemented DoG weights in Equation 4. Equation 5, therefore, provides a compact continuum description for relating the implemented recurrent kernel, event-driven input, and observed hardware regimes.

The relevant concept from Amari is *localised excitation*: a finite active region that can remain active after being triggered and can shift in response to non-uniform input. In the tracking network, this localised excitation is the target-location bump. The bump is initiated when event input pushes a small region above the threshold. Local recurrent excitation then helps the region remain active, while the broader inhibitory surround prevents the bump from expanding across the sheet.

For the analysis below, let *B*_*a*_ denote an active region of radius *a*. The recurrent drive at the edge of that region can be summarised by:


$$\begin{array}{l} M\left(a\right)=.\int_{B_{a}}w\left(∥\text{x}-{\text{x}}^{\prime }∥\right)\text{d}{\text{x}}^{\prime }{|}_{∥\text{x}∥=a}. \end{array}$$ (6)

Equation 6 provides a diagnostic for the implemented kernel: it indicates whether cells at the edge of a candidate bump receive enough recurrent support to remain active, while still allowing the inhibitory surround to contain the active region.

The recurrent drive at the centre of the same candidate bump is:


$$\begin{array}{r} W_{B_{a}}\left(\text{0}\right)=\int_{B_{a}}w\left(||{\text{x}}^{\prime }||\right)d{\text{x}}^{\prime }=2\pi \\ \left[A_{e}\sigma_{e}^{2}\left(1-e^{-a^{2}/2\sigma_{e}^{2}}\right)-A_{i}\sigma_{i}^{2}\left(1-e^{-a^{2}/2\sigma_{i}^{2}}\right)\right]. \end{array}$$ (7)

This expression makes the design intuition explicit: excitation must be strong enough to sustain the centre of the bump, while inhibition must be broad enough to contain its edges.

### 2.3 Operating regimes of the bump

The implemented network requires an operating regime in which the active region is both self-sustaining and spatially contained. We characterise this regime using two quantities from the Amari field description: the recurrent drive at the centre of the bump and the change in recurrent drive at its edge.

These quantities give two practical criteria:


$$\begin{array}{l} \underset{\text{centre receives sufficient recurrent support}}{\underset{︸}{.\underset{B_{a^{⋆}}\left(\text{c}\right)}{\int }w\left(∥\text{x}-{\text{x}}^{\prime }∥\right)\text{d}{\text{x}}^{\prime }{|}_{\text{x}=\text{c}}>\vartheta }}\;\text{and}\\ \underset{\text{edge drive decreases as the bump grows}}{\underset{︸}{M^{\prime }\left(a^{⋆}\right)<0}}. \end{array}$$ (8)

Here *a*^⋆^ denotes the candidate radius of the active bump, $B_{a^{⋆}}\left(\text{c}\right)$ is the corresponding active region centred at the bump centroid **c**, and the centre-drive condition is evaluated at **x** = **c**.

The first criterion prevents the active region from collapsing after the initial event input disappears. The second criterion prevents the active region from spreading without bound. Together, they describe the intended bump operating regime.

Equation 8 is a design criterion for the continuum field. It assumes a circular active region of a single radius, a continuous domain, an untruncated kernel, and real-valued weights. The deployed sheet is a finite lattice with open boundaries, a truncated kernel, integer weights, discrete-time updates, and a hard reset. We therefore use Equation 8 to select the deployed amplitudes and widths, and treat the dynamics of the finite network separately in Section 2.5.

These criteria also clarify the failure modes observed in hardware. If the centre drive is too weak, activity decays and the network enters a dead regime. If the edge receives too much support, activity expands, and the network enters a flood regime. Between these cases is the useful tracking regime, where activity remains compact, persistent, and locatable. This also motivates the finite local inhibitory surround. Inhibition in the deployed network is a local surround formed by the negative lobe of the difference-of-Gaussians kernel out to the truncation radius, not a global inhibitory pool. Global inhibition can select a winning location, but it tends to collapse the representation to a discrete winner. The DoG surround instead allows a small neighbourhood of cells to remain active, preserving a spatially graded estimate of the target position.

### 2.4 Tracking of a moving stimulus

For tracking, the input to the recurrent sheet is not stationary. The event-camera front end produces a target-related activity profile whose centre changes over time. This can be written schematically as:


$$\begin{array}{l} I\left(\text{x},t\right)=I_{0}\phi \left(\text{x}-\text{p}\left(t\right)\right), \end{array}$$ (9)

where ϕ is the input profile and **p**(*t*) is the target position on the sheet. In Amari's description, localised excitation can move in response to non-uniform input. In the present network, new events therefore pull the recurrent bump towards the current target location, while recurrent dynamics preserve the estimate between event updates.

Because the membrane and recurrent dynamics are not instantaneous, the bump may lag behind a moving target. This lag is expected to increase for faster target motion and to decrease when the recurrent interaction provides stronger spatial restoring drive. Predictive velocity injection is used to compensate for this effect by shifting the input slightly in the direction of motion before it is projected onto the recurrent sheet. The measured tracking experiments then test whether the recurrent bump remains localised while following the moving input.

### 2.5 The finite deployed network

Equation 8 provides a continuum design criterion, whereas the deployed network is finite, quantised, radially truncated, and updated in discrete time. We therefore analyse the exact integer-weight network independently of the continuum approximation and hardware measurements.

For an active set *S*, we track its active mass, decoded centroid, and spatial spread, with the latter used as the effective bump radius. For numerical classification, a trajectory was considered to have reached a settled stationary or periodic regime when, over 50 successive updates, the active mass and spread repeated periodically, and the centroid varied by less than 0.1 lattice cells.

In the absence of feedforward input, the recurrent drive to neurone *n* is:


$$\begin{array}{l} D_{n}\left(S\right)=\underset{m\in S}{\sum }W_{nm}. \end{array}$$ (10)

Using the recurrent drive defined in Equation 10, projecting the full LIF state onto a binary active-set representation gives the effective active-set transition map:


$$\begin{array}{l} T\left(S\right)=\left\{n:D_{n}\left(S\right)\geq \vartheta_{\text{eff}}\right\},\;\vartheta_{\text{eff}}=\left(1-\alpha \right)\vartheta =5. \end{array}$$ (11)

Equation 11 is a reduced description of recurrent recruitment and loss of activity, rather than the exact timestep update of the LIF network. All numerical finite-grid results reported below were obtained by iterating the full discrete-time LIF dynamics of Equation 2, including membrane leak, thresholding, and reset.

Quantising the DoG kernel of Equation 4 produces excitatory weights of +7 for face neighbours and +4 for diagonal neighbours, followed by an inhibitory shell of weight −1 from radius 2.24 to the truncation radius *R*_kernel_ = 4. There is no self-connection. With open boundaries, this construction yields exactly 5, 812 excitatory and 23, 832 inhibitory connections, matching the network loaded onto the chip. For compact seeds near the deployed input size, the network converges rapidly to a period-two orbit whose active mass alternates between 17 and 13 neurones, with a fixed centroid and a mean active mass of 15 neurones. The alternation is a consequence of the reset dynamics: neurones reset to *V*_reset_ = 0 after firing, causing the outer region of the bump to refresh on alternate updates. No additional refractory period is imposed. Introducing a one-timestep refractory period suppresses the recurrent core on alternate steps and causes the state to collapse within four updates.

The convergence analysis across a wider range of seed sizes shows that the operating point supports a bounded family of compact stationary or low-period states rather than a unique seed-independent orbit. Seed placement at the centre, edge midpoints, and corners preserves the decoded centroid without systematic drift in the tested cases, indicating that the sustained state is not restricted to a preferred lattice location. The active-mass oscillation remains internal to the recurrent state because the readout decodes the centroid, which is stable across the orbit. Section 4.2 compares these finite-grid dynamics with the measured hardware operating corridor.

### 2.6 Mapping to hardware parameters

The continuum widths relate to the discrete radii through σ_*e*_≈*R*_*e*_/2 and σ_*i*_≈*R*_*i*_/2, with σ_*e*_ = 1.0, σ_*i*_ = 2.0, *A*_*e*_ = 18, and *A*_*i*_ = 4 in integer weight units, and the kernel radially truncated at *R*_kernel_ = 4. The leak factor α = 0.5 fixes τ_*m*_ = 2ms through (3). Under this short time constant, the steady-state membrane for a constant per-step drive *M* is *M*/(1−α) = 2*M*, so the effective threshold in per-step boundary drive units is ϑ_eff_ = ϑ/2. With ϑ = 10, this places the stable bump radius near two lattice cells, since the quantised boundary drive crosses ϑ_eff_ with a negative slope there. The feedforward input weight is set strictly above the threshold, *I*_max_ = 15>ϑ, so that a coincident injected seed cluster reliably nucleates a bump, whereas a single stray input cell cannot. All parameters are listed in Tables 1 and 2.

**Table 1:** Network parameters and their quantised hardware counterparts on the SpiNNaker 2 chip.

Symbol	Meaning	Value
*W*×*H*	Sheet dimensions (neurones)	32 × 24 = 768
*A*_*e*_	Excitatory amplitude	18
*A*_*i*_	Inhibitory amplitude	4
σ_*e*_	Excitatory width	1.0
σ_*i*_	Inhibitory width	2.0
α	Leak factor	0.5
τ_*m*_	Membrane time constant	2ms
ϑ	Firing threshold	10
*V*_reset_	Reset potential after a spike	0
*I*_max_	Feedforward input weight	15
*R*_kernel_	Kernel truncation radius	4
sheet boundary condition		Open

Weights are in integer units; the kernel is radially truncated at *R*_kernel_ = 4.

**Table 2:** Front-end, closed-loop, predictor, gating, and classifier parameters, completing the specification begun in Table 1.

Symbol	Meaning	Value
Field and input		
Ω	Spatial domain of the field, Equation 5	[0, *W*) × [0, *H*)
*R*_*e*_	Discrete excitatory radius, σ_*e*_≈*R*_*e*_/2	2
*R*_*i*_	Discrete inhibitory radius, σ_*i*_≈*R*_*i*_/2	4
*I*_0_	Input amplitude, Equation 9	*I*_max_ = 15
ϕ	Input profile, Equation 9	2 × 2 cluster
Perception front end		
Δ_acc_	Event accumulation window	10 ms
β	Centroid EMA weight, Equation 12	0.35
	Noise floor, absolute	20 events/cell
	Noise floor, relative	3.0 × running mean
	Minimum active neighbours	2
	Per-cell refractory mask	1 window
Closed-loop, prediction, and gating		
	Readout period	20 ms
*R*_gate_	Spatial gating radius	6
*w*_pred_	Predictor weight, sub-threshold	6
*T*_pred_	Predictor look-ahead	120 ms
*R*_pred_	Cells pre-excited at the predicted centroid	1
τ_*v*_	Velocity estimator time constant	60 ms
	Refractory mechanism, sheet neurones	reset to *V*_reset_
Regime classifier, Section 3.5		
[*n*_lo_, *n*_hi_]	bump_ok active-count band	[6, 24]
ρ_max_	Spread limit	3.0
dead	Sub-threshold residual state	|*S*| < *n*_lo_
bump_ok	Count in band, spread within limit	*n*_lo_ ≤ |*S*| ≤ *n*_hi_, ρ ≤ ρ_max_
diffuse	Count in band, spread above limit	*n*_lo_ ≤ |*S*| ≤ *n*_hi_, ρ>ρ_max_
flood	Count above band	|*S*|>*n*_hi_

Positions and radii are in lattice cells.

### 2.7 Architecture for bump tracking

Figure 2 shows how the theoretical picture maps onto the implemented system. The event camera and front end produce a spatial activity map of the target on the same 32 × 24 grid as the SpiNNaker event layer. Active grid cells inject spikes into corresponding locations on the recurrent neural field. Local excitation recruits neighbouring cells around the input, and the broader inhibitory surround suppresses distant activity, forming a compact bump centred on the target evidence. The important architectural distinction is that the event input provides current sensory evidence, while the recurrent neural field provides state. The input map can be noisy, sparse, or briefly absent, but the bump is maintained by on-chip recurrence between updates. The tracking readout therefore receives a stabilised state estimate rather than a raw event centroid. In this sense, the network uses Amari's localised-excitation principle as an engineering mechanism for event-based tracking: the target position is represented by the state of a recurrent dynamical field, not by a feedforward detection alone.

**Figure 2:**
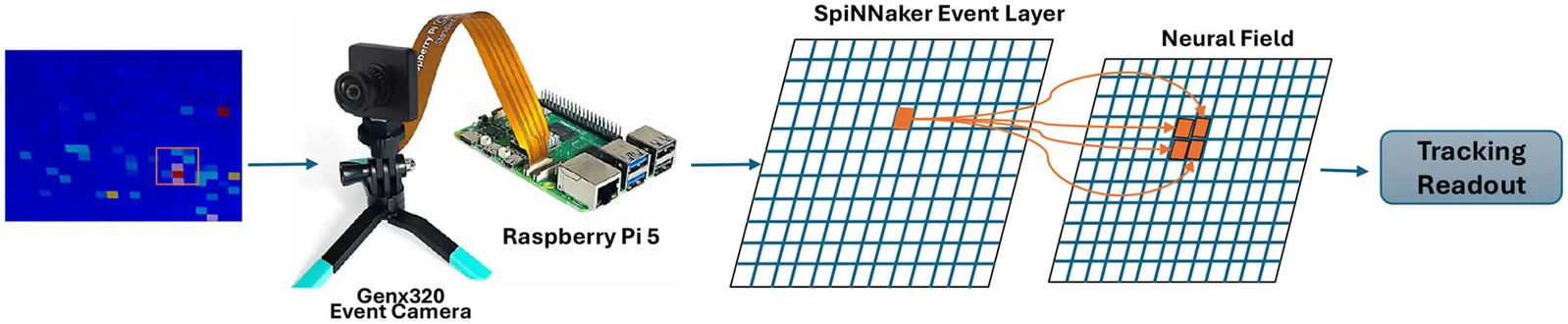
Pipeline from event camera to tracking readout. The Raspberry Pi 5 extracts a target cluster from raw events and injects it as spikes into the SpiNNaker 2 neural field, where recurrent DoG connections stabilise a localised bump at the target location.

## 3 System architecture and methods

The theoretical design is implemented as the three-stage loop in Figure 1: event-based perception on the Raspberry Pi 5, recurrent state estimation on SpiNNaker 2, and host-side motor mapping. This separation keeps the recurrent memory and stabilisation on the neuromorphic device while retaining a lightweight front end for sensor interfacing and experimental control.

### 3.1 Overview of the closed-loop

A Prophesee GenX320 event camera observes the rendered target and streams ON and OFF events to a Raspberry Pi 5. The front end converts each accumulation window into a target estimate on the same 32 × 24 grid as the neural sheet and sends the active cell identities to a single-chip SpiNNaker 2 board. The recurrent network maintains a localised bump, which is read back as a continuous coordinate and passed directly to the guidance controller. The host therefore performs sensor preprocessing, communication, and motor mapping, whereas bump formation, persistence, and stabilisation occur on-chip.

### 3.2 Event camera and perception front end

The event camera produces asynchronous ON and OFF events. The host front end accumulates events into short temporal windows of Δ_acc_ = 10ms, giving an update rate of 100Hz, and bins them onto the 32 × 24 grid that matches the neural sheet. Each window passes through a four-stage filter. First, an adaptive per-cell noise estimate is maintained as an exponential moving average of the event count, and a cell is admitted only if its count exceeds both an absolute floor and a multiple of its noise estimate, which suppresses sensor background and flicker. Second, a spatial coherence pass removes isolated active cells that have too few active neighbours, since a genuine moving object produces a spatially contiguous response. Third, a short refractory mask prevents the same cell from dominating consecutive windows.

The final stage produces the target coordinate supplied to the attractor. Because a moving object generates events around its edges, selecting the densest cell or blob can make the estimate jump between different parts of the object. We instead compute the event-weighted centre of mass of all surviving cells and smooth it across windows with an exponential moving average,


$$\begin{array}{l} \hat{\text{c}}\left(t\right)=\beta {\text{c}}_{\text{raw}}\left(t\right)+\left(1-\beta \right)\hat{\text{c}}\left(t-{\text{Δ}}_{\text{acc}}\right),\;\beta =0.35, \end{array}$$ (12)

where **c**_raw_(*t*) is the instantaneous centre of mass. A fixed 2 × 2 cluster centred on $\hat{\text{c}}\left(t\right)$ is transmitted to the chip. Each packet contains a sequence number, a send timestamp for latency measurement, the smoothed centre, and the cluster cell identities.

### 3.3 On-chip spiking attractor network

The network on the chip comprises 768 leaky integrate-and-fire neurones arranged as the 32 × 24 topographic sheet of Section 2.1, plus a matched readout population and a predictor population used for velocity injection. The recurrent connectivity is the true difference of Gaussians kernel of Equation 4, evaluated for every neurone pair within the truncation radius *R*_kernel_ = 4 and quantised to integer weights, giving 5, 812 excitatory and 23, 832 inhibitory connections, which is 29, 644 recurrent synapses in total. Because the hardware represents excitatory and inhibitory synapses on separate projections, the summed kernel is split into a positive part and a negative part that together reproduce (Equation 4). The neurone and kernel parameters are those of Table 1.

Feedforward input from the perception front end is delivered to the corresponding sheet neurones with weight *I*_max_ = 15, which is above threshold so that a coincident injected cluster nucleates a bump, but which acts only as a nudge to an already established bump because of the gating described next. The readout population mirrors the attractor sheet one-to-one through above-threshold connections, so that reading the readout reports which attractor neurones are currently active.

### 3.4 Closed-loop link, gating, and decoding

The host-to-chip link uses a datagram protocol over the local network. To guarantee that the network is always driven by the most recent observation, the receiver runs a dedicated reader that drains incoming packets into a single-slot mailbox and an injector that always consumes the freshest packet. When the injector is briefly busy, older packets in the mailbox are overwritten rather than queued, so the only packets ever discarded are stale ones, and the current position is never lost to buffer overflow.

Injected input is spatially gated. Once a coherent bump exists, only input cells within a gating radius of the current bump centre are delivered to the sheet; input far from the bump is treated as spurious and rejected. As the front end already supplies a smoothed, coherent target, the gating radius is set generously so that the bump follows the full range of normal stimulus motion and rejects only genuine outliers.

The bump position is decoded once per readout window as the activity-weighted centroid of the active readout neurones, with the active count and the spatial spread computed alongside it. A short velocity estimate is maintained from the decoded centroid, and a predictor population is driven a fixed number of steps ahead along the velocity vector with a sub-threshold weight, which biases the bump in the direction of motion and cancels the leading-order tracking lag without forcing the bump position.

### 3.5 Operating regime classification

To characterise the network state online, each readout window is classified by its active count and spatial spread into one of four exhaustive regimes. DEAD denotes fewer than *n*_lo_ = 6 active neurones, including complete silence and small residual states. BUMP_OK denotes a single compact bump within the target active band and below the spread limit and represents the desired regime. DIFFUSE denotes an active count within the band but a spread above the limit. FLOOD denotes an active count above the upper band, the saturated regime in which activity fills the sheet. The same definitions were used for all 96 E2 parameter settings. These labels operationalise the three theoretical outcomes of Section 2.3; DEAD is the loss of the centre drive condition, FLOOD is the loss of the edge containment condition, and BUMP_OK is the intersection that the operating conditions of Section 2.3 predict.

### 3.6 Experimental protocol

We evaluate the system with four experiments, denoted E1 to E4, each designed to isolate a specific claim of Section 2 and building progressively from basic network characterisation to closed-loop control.

**E1, bump formation and persistence**. The purpose is to verify that the network holds genuine recurrent state on-chip, distinguishing it from a feedforward map that collapses the moment input stops. A bump is seeded by a brief feedforward input; the input is then removed, and the active neurone count is monitored over time across four repeated removal trials over a 60 s session. Having established that a stable bump exists and persists, E2 asks where in parameter space it can be found.

**E2, stability boundary**. The purpose is to verify that the analytic stability conditions of Section 2 predict the observed hardware behaviour. The excitatory and inhibitory amplitudes *A*_*e*_ and *A*_*i*_ are swept across a grid of values, and for each setting the network is classified as Dead, Bump_ok, or Flood. With the stable operating regime confirmed and an operating point selected, E3 tests whether the bump can follow a moving target.

**E3, attractor stabilisation and target tracking**. The purpose is to measure tracking accuracy and to quantify the contribution of each pipeline component to that accuracy. A star-shaped object orbits continuously at three prescribed angular velocities, and the decoded bump centroid is compared against the ground-truth object position logged by the simulation. The resulting readout windows are repeated observations within one continuous hardware session and are not treated as independent experimental replicates. The experiment is repeated under three ablated configurations, removing prediction, spatial gating, and inhibition in turn. With tracking characterised, E4 evaluates hardware-in-the-loop guidance of the simulated robot.

**E4, closed-loop robot guidance**. The purpose is to demonstrate that the decoded bump position is directly usable for real-time motor control in a hardware-in-the-loop configuration without additional host-side smoothing. The bump position drives a distance-aware steering law that sends directional commands to a simulated robot orbiting continuously within camera view. Twenty trials are performed, comprising 10 recurrent and 10 matched non-recurrent trials using the same 10 start positions. The recurrent sheet is not reset between trials, so the two conditions are interleaved and paired by start position to limit bias from state carry-over.

## 4 Results

### 4.1 Bump formation and persistence (E1)

When input is supplied and then withdrawn, the bump does not vanish with the input but persists on the sheet, sustained by recurrent excitation. A memoryless feedforward map driven only by the current input would become inactive when that input stops. Figure 3 shows the active neurone count over a session containing 4 input-removal windows of increasing length, of 1, 2, 4, and 8 s. A staircase of window lengths is used in preference to repeated windows of one duration because the bump surviving each rung in turn is visible directly, and because a front end that leaks events during a removal would reveal itself on a short rung before a long one had been spent.

**Figure 3:**
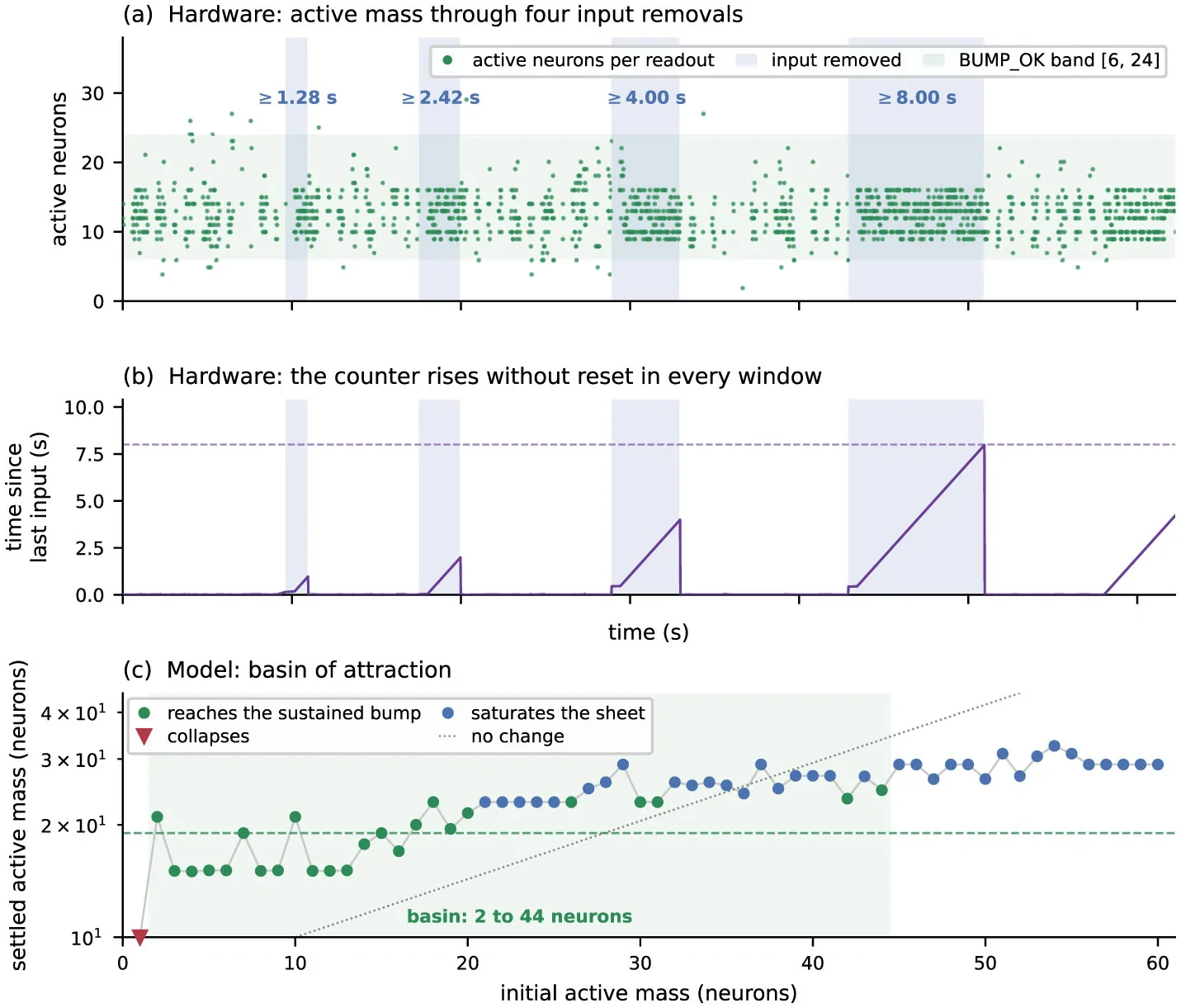
Bump persistence after input removal. **(a)** Active neurone count; shaded windows mark the four input-removal episodes, annotated with the longest interval in each without an input packet. These are lower bounds: the bump was still active at the end of every window. **(b)** Time since the last input packet, which rises without reset and reaches 8.00 s in the final window. (c) Basin of attraction in the finite-grid model: settled against initial active mass. Collapsed runs settle at zero and are drawn as triangles on the axis floor. **(a,b)** are hardware, **(c)** is the model.

Throughout every removal window the active count remains within the Bump_ok band, and panel (b) confirms that the time since the last input packet rises without reset in each window, which is what establishes that input genuinely stopped rather than merely thinned. The bump remained active at the end of all four windows, so the durations are lower bounds on the lifetime of the recurrent state and not measured times to collapse. The longest uninterrupted interval without input was 8.00 s, over which the active mass held at 12.5 neurones, the spatial spread at 1.34 cells, and the decoded centroid moved by at most 0.50 cells. Across the four windows, the active mass stayed between 12.2 and 12.8 neurones and the spread between 1.29 and 1.47 cells, so the state is sustained rather than slowly decaying. As the recurrent state remains stable throughout the longest observation window tested, Panel (c) instead reports survival in the finite-grid model of the same quantised network, evaluated over 240 trials spanning five inhibition amplitudes and initial conditions varying in seed size and position. At the deployed amplitudes, the sustained state is an exact period-two orbit of the deterministic update map and never collapses, which is consistent with the hardware observation. Collapse appears only once the inhibitory amplitude is raised above the corridor in 21% of trials at *A*_*i*_ = 6, 71% at *A*_*i*_ = 7 and every trial at *A*_*i*_ = 8.

The measured persistence demonstrates that the implemented network exhibits genuine on-chip recurrent dynamics rather than behaving as a feedforward mapping with host-side smoothing, as only a recurrent attractor can maintain a localised activity bump after sensory input is removed.

### 4.2 Hardware obeys the analytic stability boundary (E2)

Sweeping the kernel amplitudes across a grid of excitatory and inhibitory values spanning the neighbourhood of the operating point reproduce the three regimes anticipated by Section 2.3. When the centre drive falls below the threshold, the bump collapses to Dead; when the inhibitory surround is too weak to contain the edge, the sheet saturates to Flood; between these extremes lies the Bump_ok corridor. Of the 96 grid points measured, 49 were classified as Bump_ok, 19 Flood, and 28 Dead. No Diffuse outcomes were observed, consistent with the expectation that the DoG kernel cannot sustain a spatially extended yet moderately active state; the same inhibitory surround that bounds the neurone count also constrains the spatial spread, so a fragmented multi-cluster configuration is suppressed by the local inhibitory surround and does not arise at steady state.

Figure 4 shows the measured outcomes on the analytic stability boundary obtained by evaluating the centre and edge conditions of Equation 8 for the quantised kernel. The Bump_ok corridor forms a diagonal band in the (*A*_*e*_, *A*_*i*_) plane, widest near the operating point and narrowing at both extremes; at low *A*_*e*_ the centre drive is insufficient to sustain the bump regardless of inhibition, and at high *A*_*e*_ the excitatory halo dominates unless inhibition is increased proportionally. The measured transitions follow the predicted curves across the interior of the swept grid, supporting the use of the continuum conditions as a predictor of the operating corridor of the deployed spiking hardware. Figure 5 then shows the corresponding transient dynamics in the exact finite-grid model. Two systematic deviations appear near the boundary, and both are physically interpretable. At low *A*_*i*_, several points the continuum model predicts as Bump_ok are measured as Flood: with weak inhibition the excitatory halo can bootstrap iteratively before the inhibitory ring forms, a transient expansion that the static analytic bound does not capture. At high *A*_*i*_ for weak *A*_*e*_, the model predicts Dead but the hardware sustains a cluster of two to four neurones: the discrete seed leaves residual excitation that the continuum disc approximation underestimates. Both deviations are confined to the boundary itself; the interior prediction, that the closed-form conditions delineate which (*A*_*e*_, *A*_*i*_) pairs yield a compact, self-sustaining bump, is supported throughout. The second deviation is reproduced independently by the finite-grid model of Section 2.5: at *A*_*e*_ = 18 with *A*_*i*_ raised to 6 or 7, the software sheet does not fall silent either but settles at a residual two to three neurones and holds there indefinitely. The residue is therefore a property of the quantised kernel rather than of the hardware. The operating point *A*_*e*_ = 18, *A*_*i*_ = 4 with threshold ϑ = 10 sits well inside the predicted corridor, away from both boundaries. At this point, the measured active count was 16 neurones with a spread of 1.58 cells, in agreement with the radius near two cells predicted in Section 2.6 and with the mean active mass of 15 neurones obtained from the full quantised LIF dynamics described in Section 2.5. Across the full Bump_ok corridor, the active count ranged from 6 to 24 neurones and spread from 0.71 to 2.26 cells, confirming that the stable regime produces compact, localised bumps throughout rather than marginal cases confined to a single parameter setting. We note that the correct diagnosis of the operating regime required reading the full state of every processing element. An early readout that decoded only the first 32 bits per element produced a position estimate confined to a row band of the sheet and masked the true active count, causing the measured regime to appear inconsistent with the theory. Correcting the readback to cover every neurone revealed the genuine regime and was necessary for the measured outcomes to align with the analytic prediction. This is a practical reminder that on neuromorphic hardware the observation path must be validated as carefully as the network itself.

**Figure 4:**
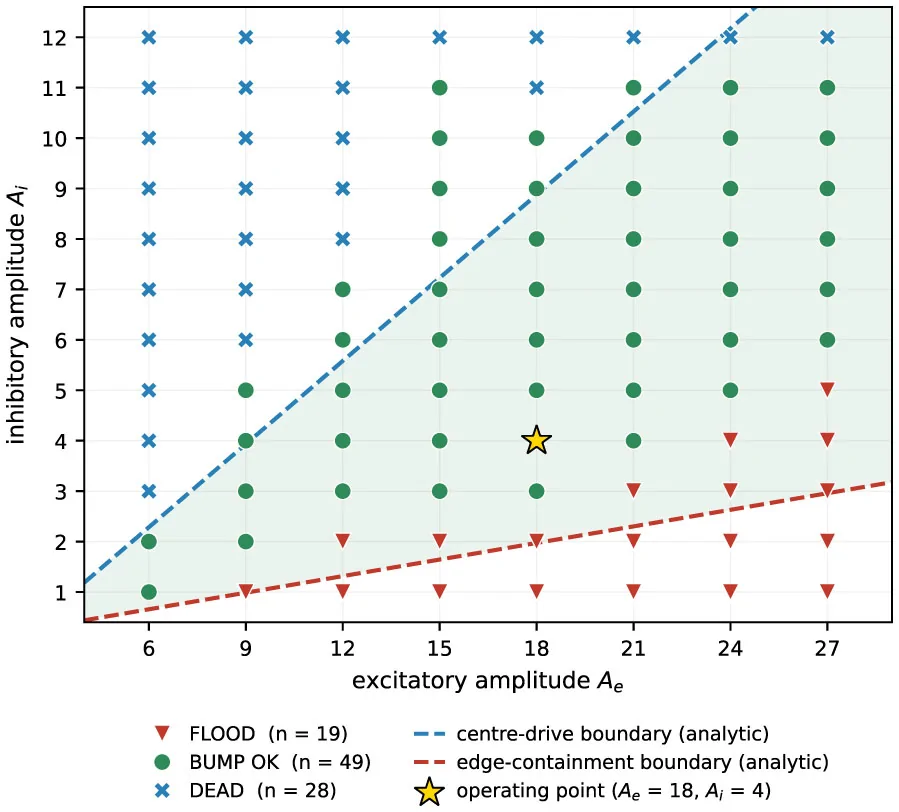
Measured hardware outcomes (DEAD, BUMP OK, and FLOOD) overlaid on the analytic stability boundary, derived from the centre-drive condition of Equation 7 and the edge-containment condition in Equation 8. The predicted BUMP OK corridor (shaded) separates the collapsed and saturated regimes. The deployed operating point (*A*_*e*_ = 18, *A*_*i*_ = 4, *andϑ* = 10) sits well within the corridor, confirming that the closed-form condition predicts the operating corridor of the spiking hardware.

**Figure 5:**
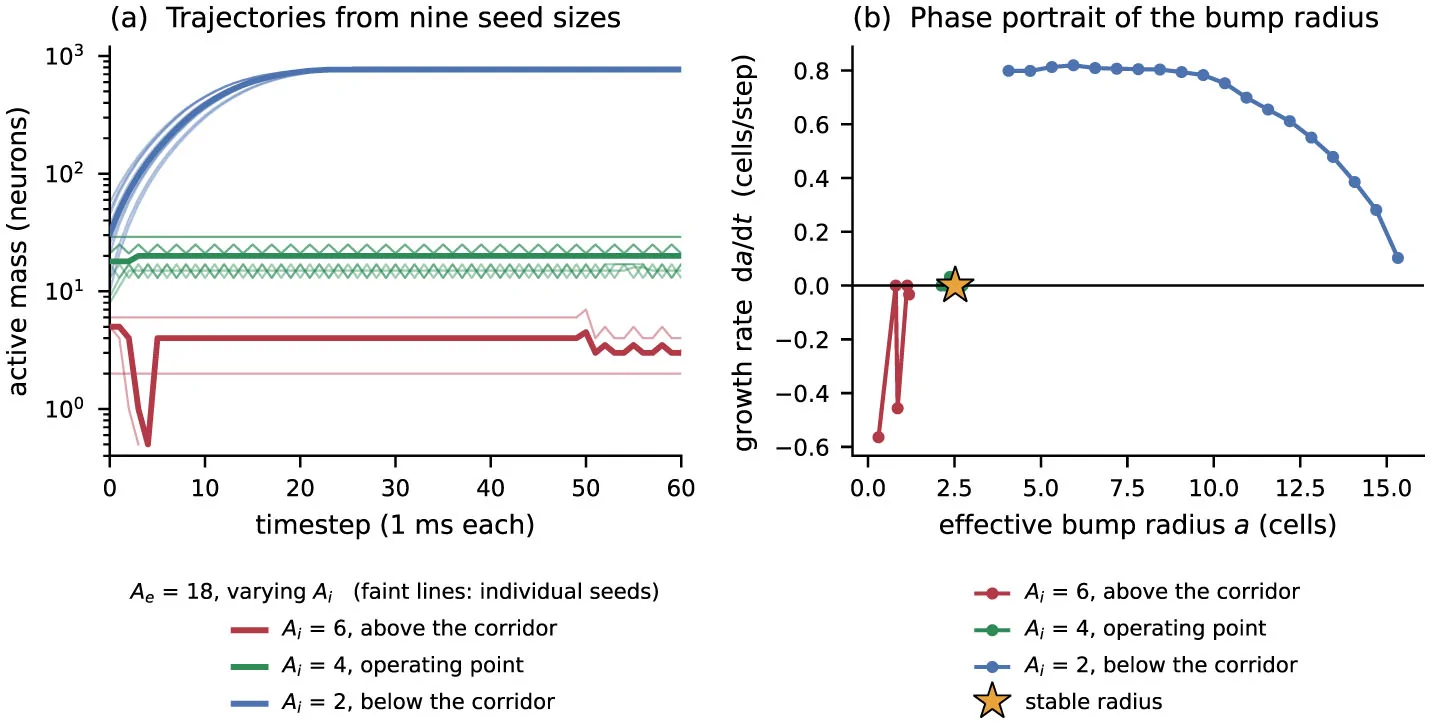
Finite-grid dynamics at *A*_*e*_ = 18. **(a)** Active mass from nine seed sizes under weak (*A*_*i*_ = 2), deployed (*A*_*i*_ = 4) and strong (*A*_*i*_ = 6) inhibition. **(b)** Corresponding radial-growth phase portrait; the deployed condition approaches a zero-growth crossing near *a* = 2.5 cells.

A categorical regime map identifies where the network settles but does not by itself show how the state approaches that outcome. Figure 5 therefore shows the transient dynamics of the exact finite-grid model at the deployed excitatory amplitude, *A*_*e*_ = 18, under weak (below the corridor), deployed (operating point), and stronger (above the corridor) inhibition. We use nine compact seed sizes at each condition. With weak inhibition (*A*_*i*_ = 2), every trajectory expands to full-sheet saturation. At the operating point (*A*_*i*_ = 4), all tested seeds remain bounded and approach stationary or low-period compact states. Stronger inhibition (*A*_*i*_ = 6) produces contraction, with most seeds collapsing and the remaining seeds settling into small residual states. The operating point, therefore, supports a bounded family of compact recurrent states rather than a unique seed-independent fixed point.

Panel (b) expresses the same dynamics through the effective bump radius *a* = ρ. The one-step radial growth rate is calculated as Δ*a*/Δ*t* = [*a*(*t*+1)−*a*(*t*)]/Δ*t* and aggregated over the sampled trajectories at comparable radii. Below the corridor, the growth rate remains positive, driving expansion; above the corridor, it is negative, driving contraction. At the operating point it approaches a zero-growth crossing near *a* = 2.5 cells. The opposite signs on either side of this crossing provide direct dynamical support for the centre-support and edge-containment interpretation, while the spread of bounded trajectories records the finite-grid and integer-weight structure that is absent from the continuum reduction.

### 4.3 Attractor stabilisation and target tracking (E3)

A star-shaped object was driven on a circular orbit through three prescribed speed phases, with angular velocity increasing from 2.6 to 4.6 rad/s. The event-camera front end supplied a smoothed centroid estimate to the chip at each 10 ms accumulation window, and the decoded bump position was compared against the ground-truth object location logged by the simulation. Figure 6 shows the bump and host-smoothed input centroid trajectories for each phase, and Table 3 reports the tracking statistics.

**Figure 6:**
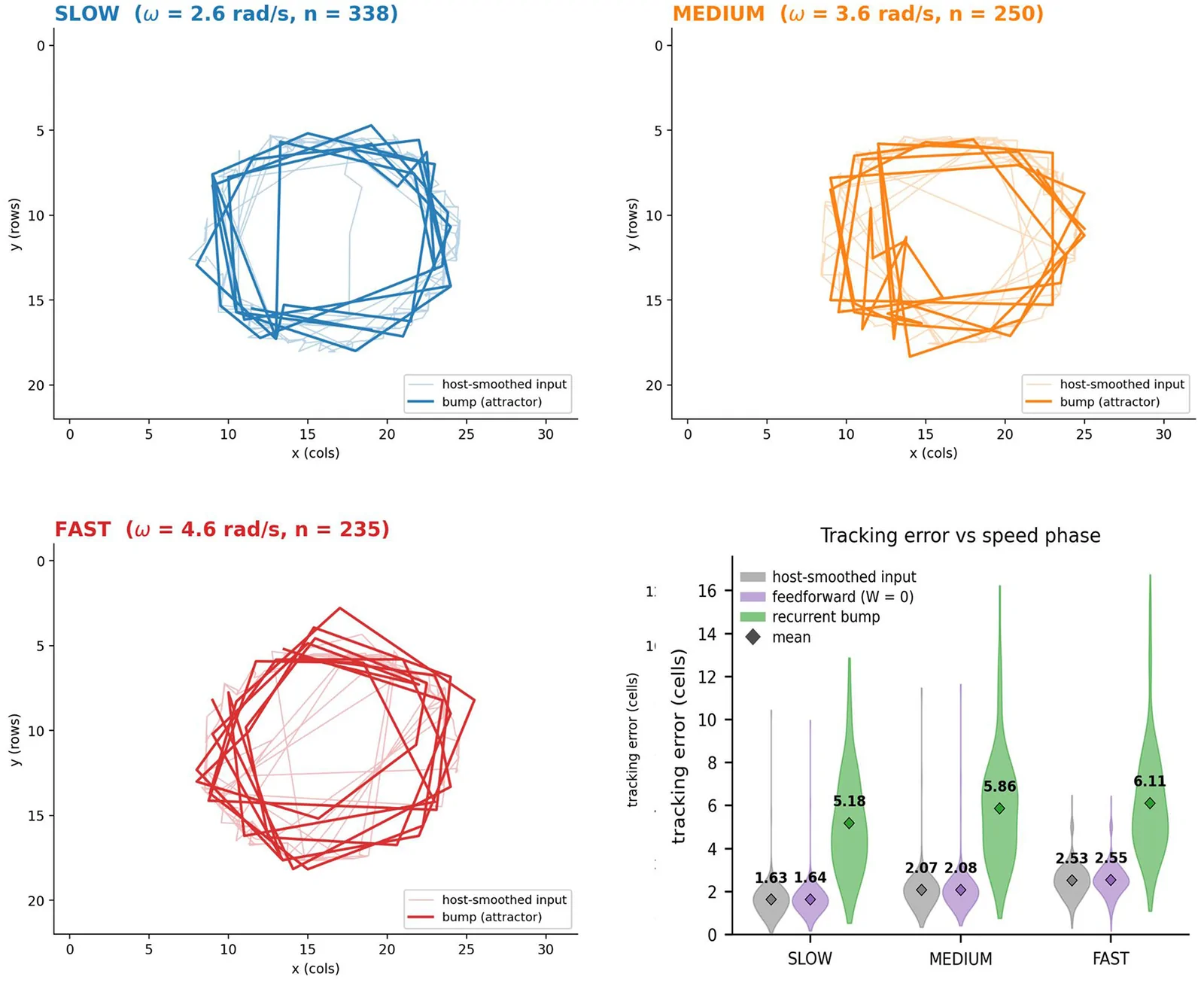
Bump centroid (solid) and host-smoothed input centroid (faded) for each speed phase. Bottom right: error against the registered object trajectory; diamonds are the mean.

**Table 3:** Tracking performance and pipeline latency.

(a) Tracking performance								
Phase	ω (rad/s)	Mean error vs. object (cells)			Lag (ms)		E2E latency (ms)	
		Host	Feedfwd	Recurrent	Host	Recurrent	Median	*P*_95_
SLOW	2.6	1.63	1.64	5.18	85	295	8.4	19.9
MEDIUM	3.6	2.07	2.08	5.86	75	190	8.2	18.8
FAST	4.6	2.53	2.55	6.11	75	180	8.5	18.1
Overall	–	2.02	2.03	5.65	75	190	8.4	19.4
***(b) Pipeline latency breakdown (ms)***								
**Stage**			**Median**			*P*_95_		
Event accumulation window			10.00			–		
Encoder filter pipeline			0.56			1.02		
UDP transfer (host to chip)			0.09			0.46		
Packet unpack			0.25			0.68		
Spike injection			0.10			0.24		
End-to-end (E2E)			8.44 ms			19.4 ms		

Errors are measured against the registered object trajectory; lags are obtained by cross-correlation. Latency statistics include only windows receiving a new input packet.

The network was classified Bump_ok in 99.6 % of all readouts windows across the full session, with a mean active count of 12.5 neurones and a mean spread of 1.56 cells, consistent with the compact bump characterised in E2 and independent of orbit speed. The simulator logs the object trajectory at 60 Hz on an independent time base. This trajectory is mapped onto the camera grid by a two-dimensional affine transform, fitted once for the rig, which resolves the mirror, scale, and offset introduced by the sensor viewpoint and leaves a registration residual of 0.58 cells. Errors below are quoted against this reference. The transform is fitted jointly with a perception delay, because the front-end estimate necessarily trails the object and absorbing that delay into the geometry would both distort the registration and conceal the delay from the measurement.

Isolating the contribution of the recurrence requires a control that shares the same input. We therefore compare four estimates of the target position against the same registered trajectory: the event-weighted centre of mass before the front-end moving average, the host-smoothed centroid that is actually transmitted to the chip, the same spiking sheet with its recurrent weights set to zero, and the deployed recurrent network. The feedforward condition is informative because the feedforward input weight is above the threshold, so a sheet without recurrence fires exactly the injected cluster and nothing else; its decoded position is therefore the transmitted centroid quantised to that cluster, and any difference between it and the recurrent network is attributable to the recurrence alone.

Mean error against the registered trajectory is 2.06 cells for the unsmoothed centre of mass, 2.02 cells for the host-smoothed centroid and 2.03 cells for the feedforward sheet, the last two agreeing to within 0.02 cells at every speed. The recurrent network reaches 5.65 cells, rising with target speed from 5.18 cells at SLOW to 5.86 at MEDIUM and 6.11 at FAST. The recurrence therefore does not improve positional accuracy over the estimate it consumes.

The discrepancy is a timing effect rather than a spatial one. Resolving the bump error into components along and across the direction of travel gives 4.48 cells along-track against 2.48 cells across-track, whereas the front-end estimate gives 1.89 and 0.53 cells. Cross-correlation places the front-end estimate 75 ms behind the object and the bump 190 ms behind it, so the recurrent stage adds 115 ms; at 3.6 rad/s on a 140 px orbit, that delay corresponds to approximately 4.5 cells of arc, which accounts for the greater part of the gap. The added delay is speed-dependent: 210 ms at SLOW, 115 ms at MEDIUM, and 105 ms at FAST. This speed-dependent reduction is consistent with predictive injection acting more strongly as the velocity estimate increases. Predictive injection fired in 99.9% of readout frames, confirming that the velocity estimator was active throughout.

Recurrence reduces frame-to-frame jitter. Evaluated over the same readout windows, the host-smoothed centroid moves by 1.57 cells between successive windows, whereas the recurrent bump moves by 1.34 cells, corresponding to a 15% reduction. The comparison includes 720 paired windows in which input and both estimates are available. These windows are repeated observations within one continuous session rather than independent experimental replicates. The recurrent estimate is smoother in 63% of the paired windows, and the Wilcoxon signed-rank statistic gives *p* = 2.5 × 10^−8^; this is interpreted only as a within-session paired comparison and not as population-level inference across independent runs. The reduction is consistent across the Slow, Medium, and Fast phases, with input-to-bump jitter ratios of 1.18, 1.20 and 1.13, respectively. The zero-recurrence control retains approximately the same frame-to-frame variation as the host-smoothed input, indicating that the reduction arises from recurrent integration rather than from spike injection or readout. The median end-to-end latency from the close of an event accumulation window to the corresponding bump update was 8.4 ms overall and below 9 ms in every speed phase, with the spike injection step itself taking tens of microseconds and the host-to-chip transport dominated by the network round trip rather than by computation. Latency is computed on readout windows in which a new input packet was received; windows falling inside an input-removal episode are excluded. Table 3 reports the full latency breakdown by stage.

### 4.4 Closed-loop robot guidance (E4)

A simulated robot moves continuously within the event camera's field of view. A localised activity bump generated by the spiking network on SpiNNaker 2 tracks the robot's position in real time, and the decoded position is used by a distance-aware steering controller to guide the robot towards a fixed goal in the bottom-left of the arena. To evaluate the functional benefit of persistent state, periodic sensor dropout is introduced. The target is hidden for 400 ms every 2.5 s while guidance continues, leaving the controller without sensory input for roughly one-seventh of each trial. Since the dropout duration is two orders of magnitude shorter than the persistence measured in E1, a network that maintains its internal state should continue guiding the robot with minimal degradation.

Two conditions are interleaved within a single session. The recurrent sheet is not reset between trials, and its state can, therefore, carry over between successive trials. Interleaving the conditions and matching them by start position limits systematic carry-over bias. The first trial begins from a cold network state and is excluded from the paired comparison. In the recurrent condition, the steering law is driven by the decoded bump. In the feedforward condition, it is driven by the host-smoothed front-end estimate, which, as established in E3, is equivalent to the same spiking sheet with its recurrent weights set to zero. The feedforward estimate expires after 40 ms without a packet; a feedforward map holds no state, so when evidence stops, it has no estimate, and allowing the host to retain the last transmitted value would give the control the very property under test. Twenty trials were run, comprising 10 recurrent and 10 matched non-recurrent trials, with each of the 10 start positions visited once under each condition. All 10 trials in each condition reached the goal. Pooled across all 20 trials, the success rate was 100%, with a Wilson 95% confidence interval of 83.9%–100%. No trial was excluded from the descriptive success-rate analysis, and none overshot the goal; the steering law's distance-dependent command rate kept the closest approach between 31.3 and 35.0 px in every trial. The network remained in the Bump_ok regime throughout, with a mean active count and spread consistent with the operating point characterised in E2.

The two conditions differ sharply, and they differ where the manipulation predicts. Paired by start position, the recurrent condition reached the goal in 7.12 s against 8.62 s for the feedforward condition, faster in all paired comparisons, a mean difference of 1.50 s and a Wilcoxon signed-rank *p* = 0.0078. The two conditions issued almost the same number of steering commands per trial, 123 against 125, and achieved almost identical path efficiency, 0.854 against 0.847. What separates them is not how much they steer but when they are able to steer at all. While the target was hidden, the recurrent condition issued 18.9 commands per trial against 6.0 (*p* = 0.0039) and closed 52.7 px of the distance to the goal against 18.4 px (*p* = 0.0039), roughly three times as much on both measures. Across the 296 readout windows in which the recurrent condition was steering with the target hidden, the bump never once collapsed; the active count averaged 12.1 neurones and never fell below 6. Over the same session, the feedforward condition had no usable estimate in 20 per cent of its readout windows, against 0.7 per cent for the recurrent condition. The persistence measured in E1 is therefore not an isolated property of the network but the mechanism by which the closed loop survives interruption of its input. Figure 7 shows the trajectories of all twenty trials, and Table 4 summarises the guidance performance.

**Figure 7:**
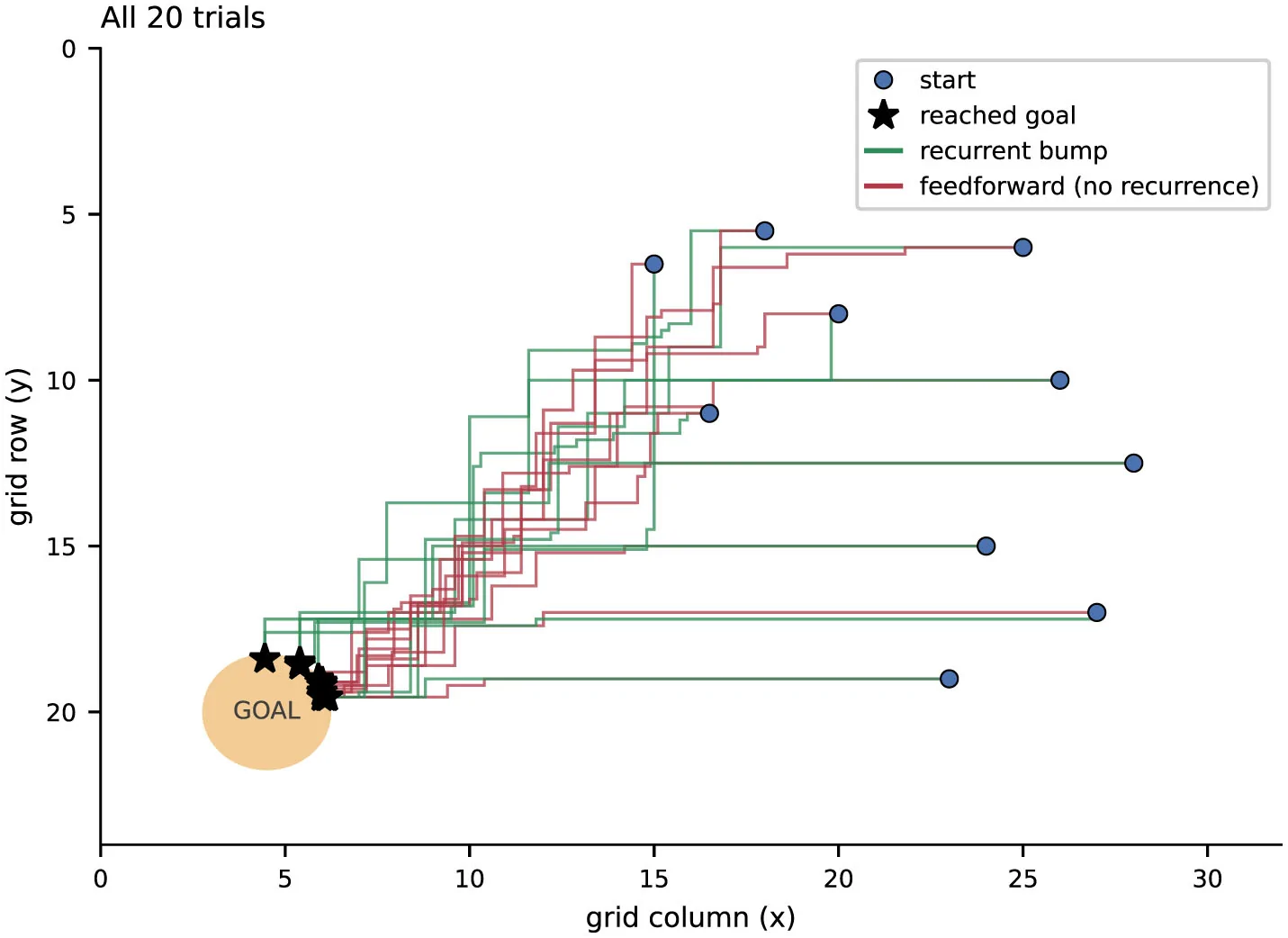
Steered trajectories for all 20 guidance trials, coloured by condition. Paths are the orbit centre, which is the quantity of the steering law moves; the robot itself orbits continuously about that centre. Circles mark trial starts, and the stars mark the arrival at the goal, shown as the orange region at the bottom left. Each of the 10 start positions is visited once under each condition.

**Table 4:** E4 closed-loop guidance under periodic sensor dropout: 10 recurrent and 10 matched non-recurrent trials, with each start position visited once under each condition.

Metric	Feedforward	Recurrent	*p*
Success rate	100%	100%	–
Mean time to goal (s)	8.62	7.12	0.0078
Mean steering commands	125	123	–
Path efficiency	0.847	0.854	–
While the target was hidden			
Commands issued	6.0	18.9	0.0039
Distance closed (px)	18.4	52.7	0.0039
Readout windows without an estimate	20%	0.7%	–

Paired comparisons and *p*-values use Wilcoxon signed-rank tests over the nine matched start positions after excluding the first trial as the single cold start. All 10 trials in each condition succeeded; the pooled Wilson 95% confidence interval across all 20 trials was 83.9%–100%.

### 4.5 Estimated power consumption

The single-chip SpiNNaker 2 laboratory board used in this study does not provide built-in board-level power telemetry. We report an indicative component-level power budget based on published values and the measured activity of the deployed network. These figures are intended to characterise the approximate operating scale of the prototype rather than to constitute a direct board-level power measurement.

The Prophesee GenX320 sensor remained in MIPI CSI-2 streaming mode throughout the experiments, for which a power consumption of 22.8 mW has been reported (Bhattacharyya et al., 2024). Its ultra-low-power monitor mode (36 μW) and deep-sleep mode were not used during active tracking. Published measurements place Raspberry Pi 5 consumption at approximately 2.7 W when idle and up to 6.8 W under computational load (Pounder and Piltch, 2023). The deployed SpiNNaker 2 network comprises 768 LIF neurones distributed across 24 processing elements. Published measurements report an efficiency of 21.59 μW/MHz per processing element at 100 MHz and 0.5 V (Höppner et al., 2021). Applying this processing-element figure to the deployed mapping and scaling of the dynamic contribution by the measured mean network activity, approximately 1.6% of neurones active per 1 ms timestep, gives an indicative on-chip active-power estimate of approximately 15 mW. The corresponding loaded-but-quiescent estimate is approximately 5 mW. This calculation assumes that the activity-dependent contribution scales approximately with the measured network activity and does not include board-level conversion losses, communication-interface consumption or other support circuitry.

Table 5 summarises the resulting component-level estimate. The Raspberry Pi 5 dominates the present prototype power budget, while the neuromorphic component remains in the milliwatt range under the stated assumptions.

**Table 5:** Estimated component-level power budget.

Component	Idle (W)	Active (W)
Raspberry Pi 5	2.70	6.80
Prophesee GenX320	0.003	0.023
SpiNNaker 2	0.005	0.015
Estimated total	2.71	6.84

Raspberry Pi 5 and GenX320 values are taken from published measurements (Pounder and Piltch, 2023; Bhattacharyya et al., 2024); SpiNNaker 2 values are activity-scaled estimates derived from published processing-element measurements (Höppner et al., 2021) and are not direct board-level measurements.

### 4.6 Ablation study

To isolate the role of each component, we evaluate three ablated configurations under the E3 protocol (Table 6). The results distinguish the mechanisms required to sustain a bounded recurrent state from those that improve tracking accuracy. Removing the inhibitory surround (*A*_*i*_ = 0) eliminates edge containment and drives the network into the Flood regime. Activity expands across the sheet, and tracking fails, confirming that the inhibitory component of the DoG kernel is essential for maintaining a compact bump.

**Table 6:** Each row removes one component from the full system and reports the effect on tracking performance.

Configuration	BUMP OK	Tracking error (cells)			Overall	Spread
	(%)	SLOW	MED	FAST	mean	(cells)
Full system	99.6	2.75	3.42	3.35	3.19	1.56
No prediction	98.3	3.24	3.60	3.74	3.53	1.60
No spatial gating	99.4	3.46	3.61	3.84	3.64	1.55
No inhibition	0.0	FLOOD				~11.0

Tracking error here is the mean Euclidean distance between the decoded bump centroid and the front-end target estimate. Absolute error against the registered object trajectory is given in Table 3.

By contrast, spatial gating and predictive injection are not required for bump formation, but both improve tracking performance. Without spatial gating (*R*_gate_ → ∞), the network remains predominantly within the Bump_ok regime (99.4%), while the mean tracking error increases by 0.45 cells (14%). The largest degradation occurs at the Slow speed (26%), where spurious inputs can influence the bump over a longer interval. Disabling predictive injection (*w*_pred_ = 0) reduces the Bump_ok rate to 98.3% and increases the mean tracking error by 0.34 cells (11%). Its effect is most pronounced at the Fast speed, where the error increases by 12%, consistent with the prediction compensating for motion-dependent lag. These results establish a clear hierarchy of function. The inhibitory surround is necessary for bounded recurrent activity, whereas spatial gating and predictive injection refine the target estimate by rejecting inconsistent input and reducing speed-dependent tracking delay.

## 5 Discussion

This study links continuum neural-field design, finite quantised dynamics and hardware behaviour within a complete event-driven guidance system. The principal contribution lies in implementing a recurrent continuous attractor on a single SpiNNaker 2 chip and demonstrating its use as a persistent state between event-based sensing and closed-loop control.

Across all excitatory—inhibitory parameter combinations, the measured Dead, Bump_ok, and Flood regimes followed the predicted operating corridor through the interior of the parameter grid. The finite-grid analysis further showed distinct transient dynamics across representative inhibition regimes: weak inhibition produced expansion to saturation, stronger inhibition produced contraction or collapse, and the deployed operating point supported bounded compact stationary or low-period states. Deviations near the analytical boundaries are attributable to effects excluded from the continuum approximation, including integer quantisation, open boundaries, transient recruitment, and reset-dependent dynamics. At the deployed operating point, the bump remained compact for at least 8 s after input removal, with no collapse observed during the longest test interval. This result establishes that the target state is maintained by on-chip recurrence rather than by continued sensory input. During tracking, recurrence reduced frame-to-frame jitter by 15% relative to the host-smoothed centroid but introduced an additional lag of 115 ms. The corresponding increase in tracking error was predominantly along the direction of motion, reflecting the inherent trade-off between persistence and responsiveness. Predictive injection reduced speed-dependent delay, while spatial gating limited the influence of inconsistent input. The guidance results demonstrate the functional value of this persistent representation. All 10 recurrent and all 10 matched non-recurrent trials reached the goal. The recurrent controller reached it 1.50 s sooner on average and maintained steering commands during the imposed 400 ms sensor dropouts. The advantage therefore arose from continued estimate availability rather than a higher command rate. Ablation further confirmed that the inhibitory surround is necessary to prevent saturation, whereas spatial gating and predictive injection primarily improve tracking accuracy.

The present implementation remains limited by the 20 ms readout interval, host-side decoding, and the spatial resolution of the 32 × 24 neural sheet. The Raspberry Pi front end also dominates the estimated power budget, and the SpiNNaker 2 contribution is derived from published chip measurements because the laboratory board does not provide built-in board-level power telemetry. Future implementations should move more of the perception and decoding pipeline onto neuromorphic hardware, increase map resolution, and evaluate direct board-level power using an external instrumented supply. A matched comparison with a conventional frame-based camera and CPU pipeline is also required to quantify any system-level performance and energy advantages. Previous event-camera systems on SpiNNaker relied on earlier sensors, larger machines, or dedicated interface hardware (D'Angelo et al., 2022; Ghosh et al., 2022; Romero Bermudez et al., 2023), while reported SpiNNaker 2 control studies used simulated sensory input (Arfa et al., 2025). The present system combines a physical GenX320 front end, a single-chip recurrent attractor, finite-network stability analysis, registered tracking evaluation, and hardware-in-the-loop guidance in one implementation.

## 6 Conclusion

A recurrent spiking continuous attractor network was implemented on a single SpiNNaker 2 chip and integrated with an event-camera front end for target tracking and hardware-in-the-loop robot guidance. The continuum analysis identified the operating corridor, while the finite-grid model and hardware sweep confirmed bounded compact dynamics at the deployed operating point. The recurrent bump persisted for at least 8 s after input removal and reduced frame-to-frame jitter by 15%. Inhibition was necessary to prevent saturation, whereas spatial gating and predictive injection improved tracking accuracy. The complete system successfully guided the simulated robot in all the trials and maintained a usable target estimate during sensor dropout.

## Data Availability

The datasets presented in this study can be found in online repositories. The names of the repository/repositories and accession number(s) can be found below: https://github.com/aitsam12/Spiking-Continuous-Attractor-on-SpiNNaker-2-.
